# Levels of Metals in Kidney, Liver, and Muscle Tissue and their Influence on the Fitness for the Consumption of Wild Boar from Western Slovakia

**DOI:** 10.1007/s12011-016-0884-z

**Published:** 2016-11-03

**Authors:** Jozef Gašparík, Łukasz J. Binkowski, Andrej Jahnátek, Peter Šmehýl, Milan Dobiaš, Norbert Lukáč, Martyna Błaszczyk, Magdalena Semla, Peter Massanyi

**Affiliations:** 1 Department of Poultry Science and Small Animal Husbandry, Slovak University of Agriculture, Trieda Hlinku 2, 949 01 Nitra, Slovakia; 2 Institute of Biology, Pedagogical University of Cracow, Podbrzezie 3, 31-054 Krakow, Poland; 3 Department of Economics, Slovak University of Agriculture, Trieda Hlinku 2, 949 01 Nitra, Slovakia; 4 Department of Animal Physiology, Slovak University of Agriculture, Trieda Hlinku 2, 949 01 Nitra, Slovakia

**Keywords:** Game, Pollution, Cadmium, Lead, Mercury

## Abstract

Due to environmental pollution, wild animals are exposed to various pollutants. Some game animals, such as wild boars are used by people for food, but their meat is not evaluated regarding pollution transfer, since they are unavailable on the official market. The aim of this paper is to present the concentrations of chosen metals (Cd, Co, Cu, Hg, Pb, and Zn) in the kidneys, liver, and muscles of wild boars (*n* = 40) hunted in eastern Slovakia, as derivatives of physiological distribution and anthropogenic pollution. We found that sex was not a statistically significant factor for metal concentrations. Tissue differences were observed for all the metals studied except for Co. Cd, Cu, and Hg showed the highest median concentrations in kidney tissue with the lowest in muscle tissue (2.73, 3.78, and 0.061 μg/g w.w., respectively). The highest Zn median concentration was noted in the liver tissue with the lowest in muscle tissue. Co and Cu concentrations varied according to the age groups. Correlations between metal concentrations in muscle and kidney tissue were not especially strong; such relationships were not found in liver tissue. Among all the potential relationships of the given metal concentrations between tissues, the only significant relationship, albeit weak, was noted for Pb in muscle and liver tissue. The concentrations found seem not to be extremely high, but according to EU maximum permitted residue levels for Cd and Pb concentrations in meat, none of the samples studied was fully fit for human consumption. TWI and risk was also excessive for both metals.

## Introduction

Many communities in many parts of the world use the wild animals as a major ingredient in their diet [1, 2]. Such animals are believed to be a source of natural meat of the highest quality. Apart from the issues of excellent growing conditions, free, as it were, from farming and medical treatment, they feed in the environment and their food is free from additives such as drugs, antibiotics, and hormones in the form of supplements [3, 4]. However, the increasing damage to the environment exposes these animals to numerous pollutants, such as metals, pesticides, PCBs and others [5–9]. Exposure is influenced not only by general pollution, but also by the animals’ position in the trophic net, their behavior, and their type of habitat [10].

One animal which is crucial in this way in Europe and Asia is the wild boar. The wild boar is a wide-spread and abundant omnivore [11–13]. It has an excellent ability to adapt to anthropogenically changed environments and, as well as the countryside, inhabits suburbs and towns [14, 15]. Due to this, wild boars’ contact with various sources of a variety of pollutants is significant. In many countries, the recent increase in wild boar populations has given rise to an intensification of hunting (also imposed by agriculture), which the consumption of boar meat has also increased [16, 17].

Metals, such as cadmium, mercury, lead, and zinc are inextricably linked to anthropogenic activity [18]. Threshold concentrations of some of these metals are regulated in meat and livestock products [19]. Metal concentrations were also measured in the tissue of some wild animals, including wild boars from various parts of the world [20–22]. Due to the fact that exposure is influenced locally, however, no average values can be given for larger areas. Since most part of the wild boar meat is not acquired via the official market, no constant monitoring of levels of potential pollutions in the meat is being carried out, including eastern Slovakia. There is thus an urgent need to present the concentrations obtained in various areas in order to verify the risk of potential consumers. Such data may additionally be used in biomonitoring evaluation of the collection areas [22–24].

The main aim of this paper is to investigate the concentrations of selected metals some of which reflect the industrial impact on the ecosystem (cadmium (Cd), cobalt (Co), copper (Cu), mercury (Hg), lead (Pb), and zinc (Zn)) in the kidney, liver, and muscle tissue of wild-boars collected in southwestern Slovakia. The concentrations were evaluated regarding the potential differences due to sex, tissue, and age group. The relationships between metal concentrations in a given tissue type, as well as comparisons between tissue types, were also checked. Finally, the fitness of the organ and muscle tissues studied was evaluated according to available thresholds, and the condition of the environment was discussed.

## Materials and Methods

**Fig. 1:**
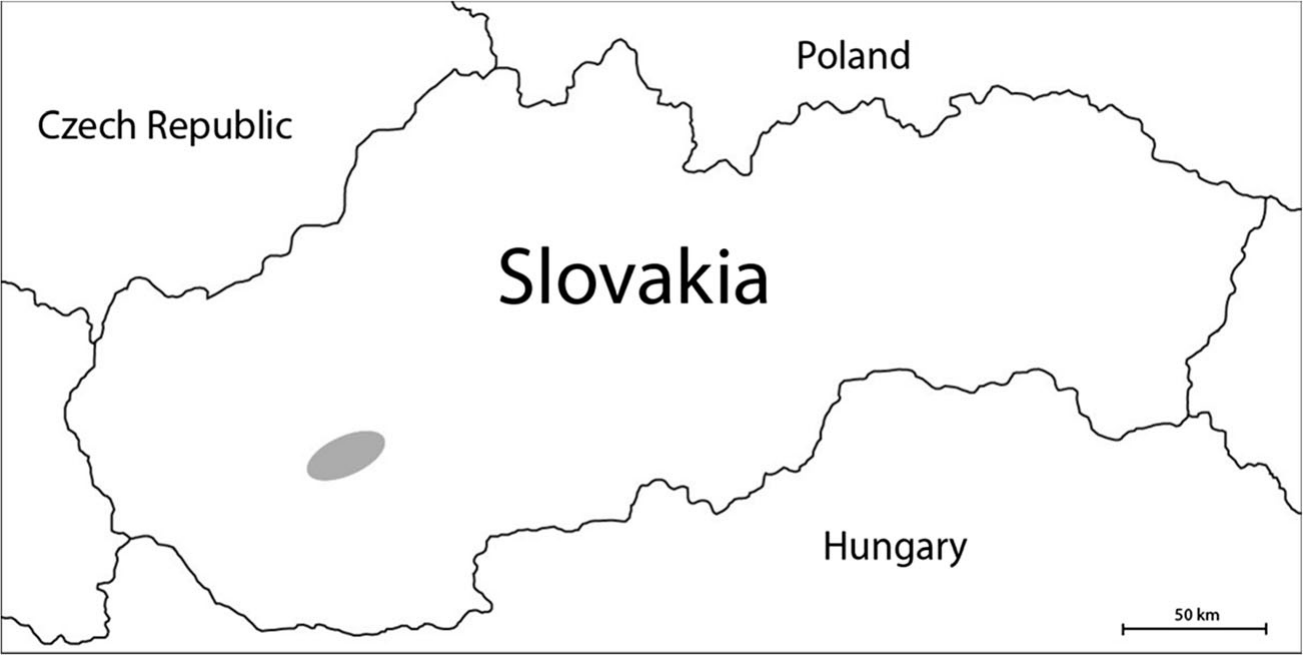
The site (*gray ellipse*) of the research—the Nitra and Topolcianky region in south western Slovakia (Europe)

Wild boars (20 females, 20 males) were collected by hunters in November and December of 2009 and 2010 in the region of Nitra and Topolcianky in western Slovakia (Fig. 1). Apart from sex, the animals were categorized according to age: 1, 2, 3, 4, and 5 years (each category with four specimens). Samples of kidney, liver, and muscle (*musculus semimembranosus*) tissue were taken, put in plastic bags, and stored at −18 °C. One to two grams wet weight (w.w.) of each sample was weighed (Mettler AE 200; accuracy to 0.0001 g) and mineralized in nitric acid (65%, SupraPUR, Merck, Darmstadt, Germany) in a microwave digestion system (Mars X) in 180 °C for 15 min and 70 °C for 20 a further minutes. The mineralized solutions were filtered (Munktell & Filtrak, no. 389, Barenstein, Germany) and made up to 50-mL flask with deionized water (Simplicity 185 Millipore SAS, Molsheim, France).

Analysis for Cd, Co, Cu, Pb, and Zn were carried out with Varian instruments: a flame atomic absorption spectrometer (SpectrAA 240 FS: Co, Cu, Zn) and an electrothermal absorption spectrometer (SpectrAA240Z: Cd and Pb). Hg concentrations were measured in fresh samples without the preparation mentioned above with a CVAAS mercury analyzer AMA-254 (Altec, Prague, Czech Republic). The final results, after comparing them to the limits of detection (Table 1), were expressed as microgram per gram w.w.

**Table 1:** Analytical parameters of methods used with the results of CRM analysis (*N* = 11)

Element	Limit of detection (in solution)	Limit of detection (in fresh sample)	Method	CRM^a^ trueness (with RSD)
Cd	10 ng/L	0.5 ng/g	ET-AAS	+8.1% (7.7%
Co	5.0 μg/L	0.25 μg/g	F-AAS	–
Cu	2.0 μg/L	0.1 μg/g	F-AAS	−1.6% (4.1%)
Hg	–	0.075 ng/g	CV-AAS	−4.7% (3.9%)
Pb	10 ng/L	0.5 ng/g	ET-AAS	+4.3% (8.1%)
Zn	0.6 μg/L	0.03 μg/g	F-AAS	−0.2% (6.1%)

^a^For Hg BCR-463 CRM was used, for other elements BCR-185R, no appropriate CRM for Co was found

For calibration and quality checks, including control solutions and spikes, a multi-standard solution CertiPUR (Merck, Darmstadt, Germany) was used. All the recoveries were satisfactory (between 90 and 110%). The BCR-185R certified reference material (CRM) was also analyzed as the final check of the whole protocol (*N* = 11) (Table 1).

### Statistical Analysis

Since the data did not meet the demands of parametric tests, we carried out the main factor robust ANOVA on ranks [25]. Age, sex, and tissue type were used as categorical factors. We used *R* Spearman correlation factors to evaluate the potential relationships between metals in the given tissues, as well as the relationships between tissues regarding the given metal. The significance level was set at 0.050. All the calculations and analyses were done with Excel 2016 for Mac (Microsoft) and Statistica 12 (StatSoft).

## Results

In all the samples, all the metals studied were present at levels above their detection limits. Since the influence of sex was not significant for all the metals studied (the lowest *p* noted for Cd was 0.060), the data was pooled regardless of sex.

**Fig. 2:**
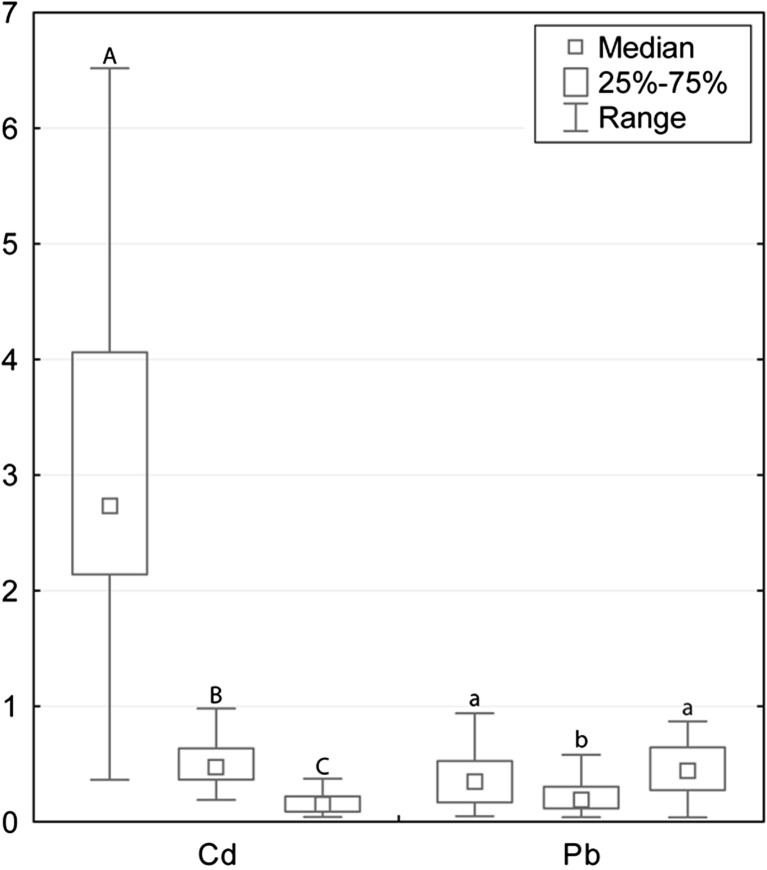
Cd, Cu, and Pb concentrations (μg/g w.w.) in the kidney, liver, and muscle tissue (consecutively) of wild boars studied. Since the factors of sex and age were not significant, the groups were merged. Different letters indicate statistically significant differences in concentrations between tissue types. Range presented for non-outlier observations

**Fig. 3:**
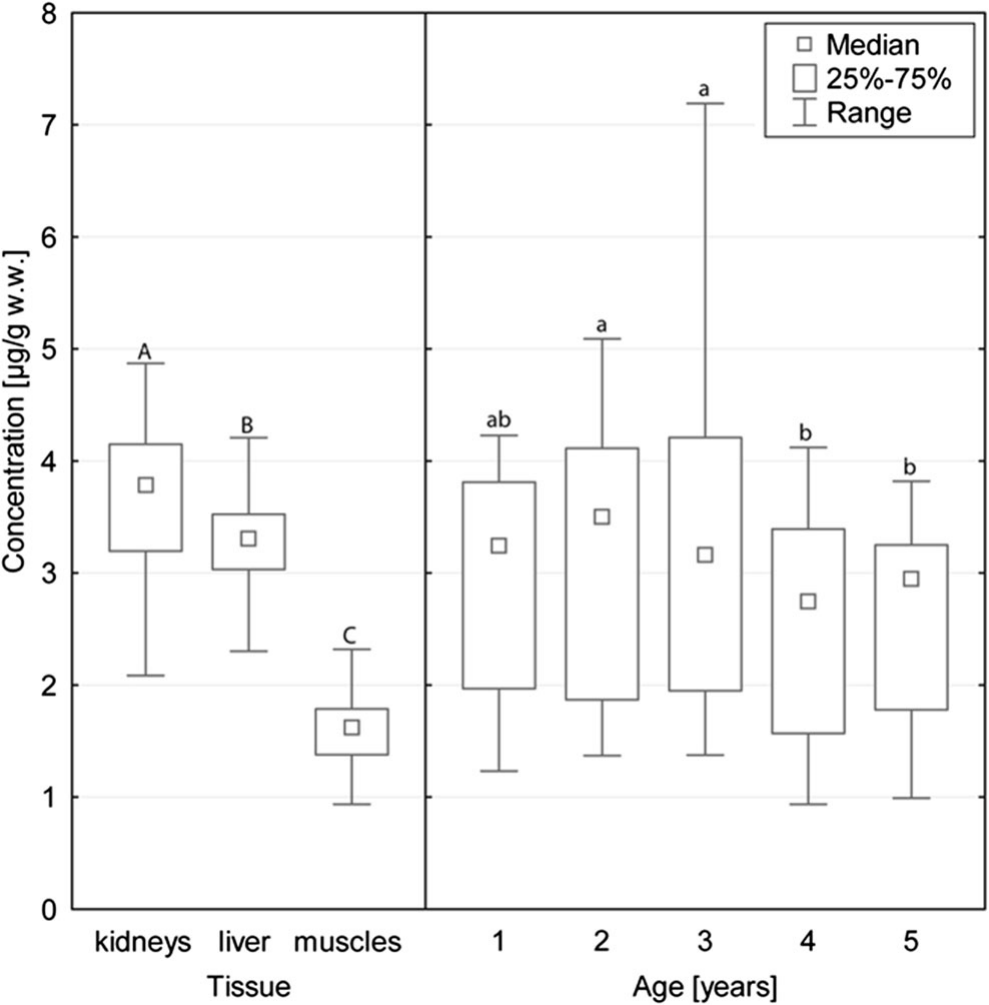
Cu concentrations in animals studied, with the significant division into tissue types and age groups studied. Different *letters* indicate statistically significant differences in concentrations between tissue types and age groups. Range presented for non-outlier observations

**Fig. 4:**
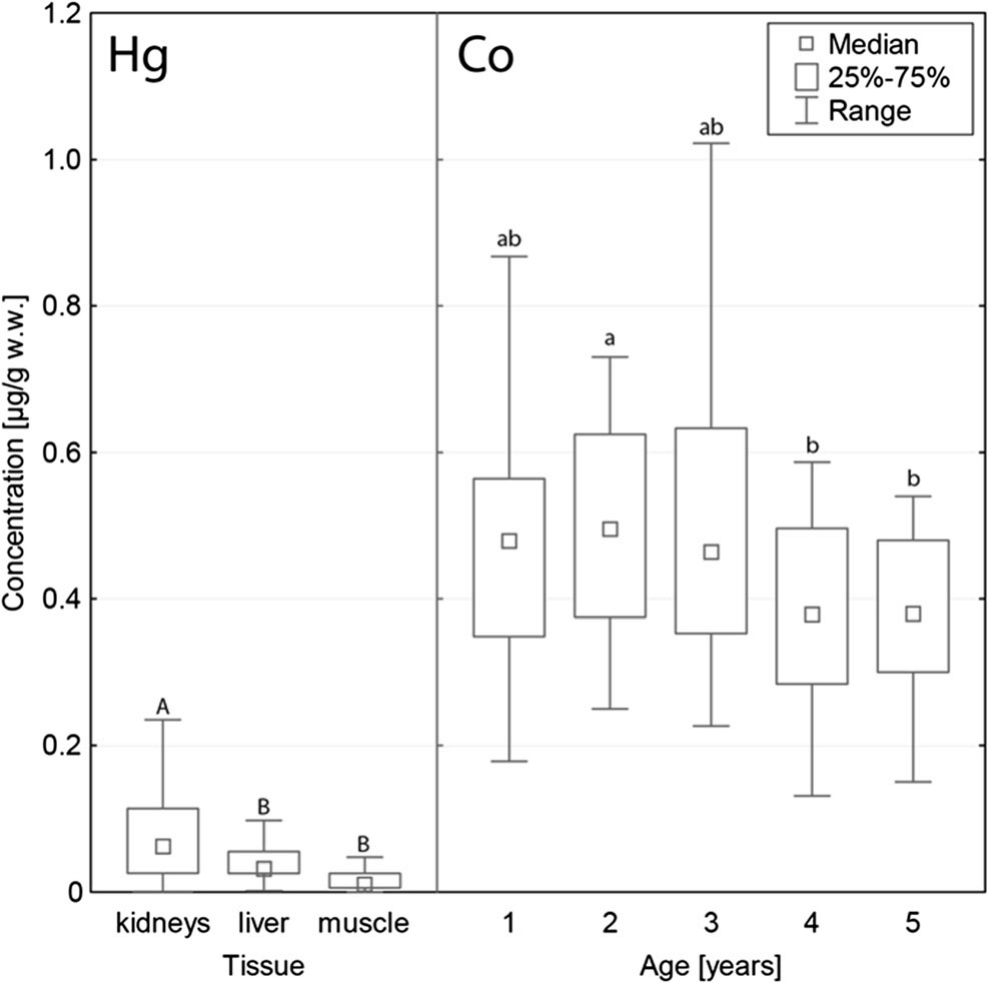
Hg and Co concentrations in animals studied. Hg concentrations differed between tissue types, but Co differed only between age groups. Different *letters* indicate statistically significant differences in concentrations between tissue types and age groups. Range presented for non-outlier observations

**Fig. 5:**
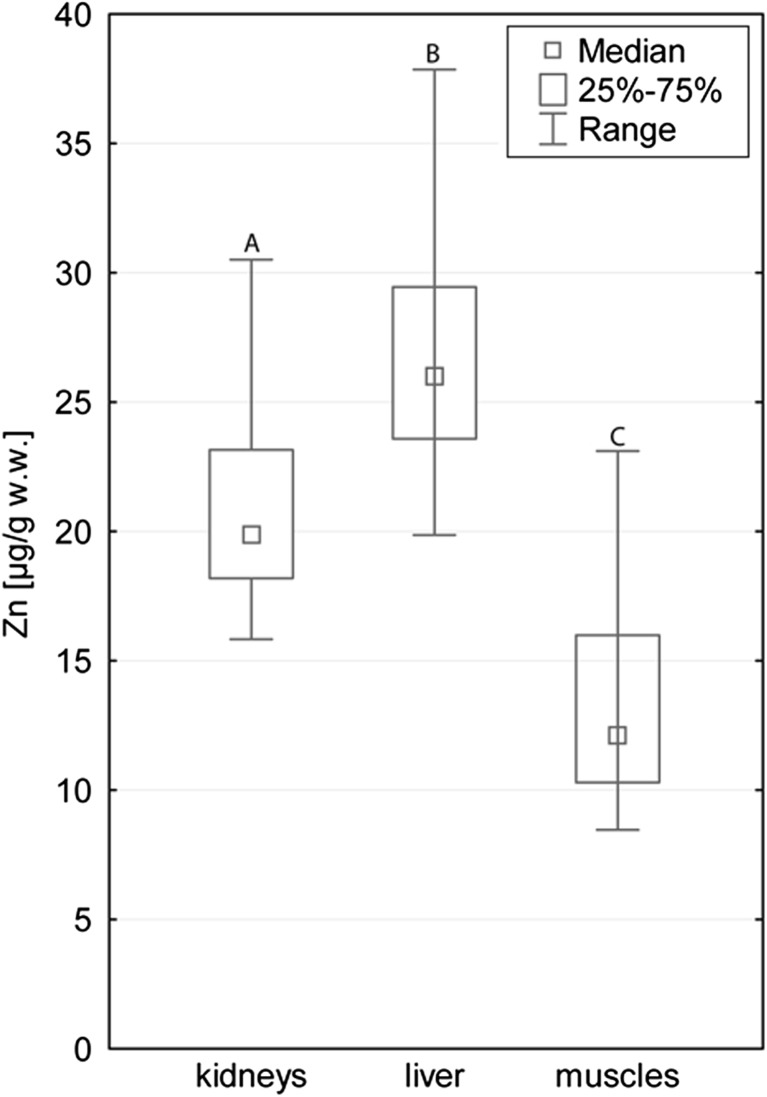
Zn concentrations in different tissue types studied of wild boar. Different *letters* indicate statistically significant differences in concentrations between tissue types studied. Range presented for non-outlier observations

The general scheme of the increasing concentrations, with some deviations between tissues, was as follows: Hg < Pb < Co < Cd < Cu < Zn. The tissue factor was statistically significant for Cd, Cu, Pb, Hg, and Zn (for all the metals *p* < 0.001) and non-significant for the concentrations of Co (*p* = 0.272). With regard to this factor, each tissue differed from others with the exception of muscle and kidney tissue for Pb, and liver and kidney tissue for Hg. Cd, Cu, and Hg had the highest median concentrations in kidney tissue (2.73, 3.78 and 0.061 μg/g w.w., respectively; Table 2); levels of concentrations in liver tissue (0.474, 3.31 and 0.031 μg/g w.w., respectively) were lower, and the lowest average concentrations of these metals were noted in muscle tissues (0.155, 1.62 and 0.011 μg/g w.w., respectively; Figs. 2, 3 and 4). The highest Zn concentrations were noted in liver tissue (26.0 μg/g w.w.), the lowest in muscle tissue (12.1 μg/g w.w.; Fig. 5). Pb occurred in the highest average concentration in muscle tissue (0.441 μg/g w.w.; Table 2), the lowest in liver tissue (0.188 μg/g w.w.; Fig. 2).

**Table 2:** Concentrations of metals studied (μg/g w.w.) in the division to tissue type (*N* = 40)

Tissue	Metal	Median	Minimum	Maximum	Q_1_	Q_3_	Range	IQR
Muscle	Cd	0.155	0.043	0.373	0.089	0.220	0.331	0.131
Muscle	Cu	1.62	0.935	2.51	1.38	1.79	1.57	0.41
Muscle	Hg	0.011	0.000	0.251	0.006	0.026	0.251	0.020
Muscle	Pb	0.441	0.039	61.3	0.274	0.645	61.3	0.371
Muscle	Zn	12.1	8.46	23.1	10.3	16.0	14.6	5.7
Liver	Cd	0.474	0.190	1.92	0.365	0.635	1.73	0.270
Liver	Cu	3.31	2.10	5.86	3.03	3.52	3.77	0.50
Liver	Hg	0.032	0.003	0.113	0.026	0.055	0.111	0.029
Liver	Pb	0.188	0.040	1.29	0.116	0.305	1.25	0.189
Liver	Zn	26.0	19.9	52.7	23.6	29.4	32.8	5.9
Kidneys	Cd	2.73	0.36	8.82	2.14	4.06	8.45	1.92
Kidneys	Cu	3.78	2.09	7.19	3.20	4.15	5.10	0.96
Kidneys	Hg	0.061	0.001	0.739	0.026	0.114	0.738	0.088
Kidneys	Pb	0.345	0.049	1.10	0.169	0.526	1.05	0.357
Kidneys	Zn	19.9	15.8	31.9	18.2	23.2	16.1	5.0
All^a^	Co	0.438	0.131	1.14	0.330	0.558	1.01	0.228

*Q* quartiles, *IQR* interquartile range

^a^Since Co concentrations did not differ between tissues, they are presented without the division

Concentrations of Co (*p* = 0.008) and Cu (*p* < 0.001) varied between the age groups studied. Generally, the highest concentrations were observed in animals of 2 and 3 years old (Fig. 3, 4). For both metals, the statistically significant differences occurred between 2-year-old animals (Co 0.495 μg/g w.w., Cu 3.50 μg/g w.w.), 4-year-olds (Co 0.379 μg/g w.w., Cu 2.74 μg/g w.w.), and 5-year-olds (Co 0.390 μg/g w.w., Cu 2.95 μg/g w.w.). In the case of Cu, the additional significant differences were noted between 3-year-old animals, four-year-olds, and five-year-olds. In all the cases mentioned, the lowest concentrations were observed in older animals.

### Correlations

We observed no relationship between metals in liver tissue. Correlations in muscle and kidney tissue were rather weak, but in three cases higher than 0.500 (Table 3).

**Table 3:** *R* Spearman correlation factors (higher than 0.5 or lower than -0.5) noted between metals in tissue studied

Tissue	Metals	*R* Spearman
Muscles	Cu–Zn	0.618
Kidneys	Cd–Pb	−0.564
	Cu–Zn	0.514

In the case of relationships of the given metal concentrations between tissues, the only significant relationship was noted for Pb in muscle and liver tissue (*R* Spearman 0.448). The generally known relationship of cadmium concentrations in liver and kidney tissue was not observed in the study (*R* Spearman 0.110).

## Discussion

We found that the sex factor had no influence on the levels of the metals studied, but the concentrations were differentiated by tissue (Cd, Cu, Pb, Zn, Hg) and age group (Co and Cu). No unanimous scheme of accumulation for metals and for tissue type was observed. Correlations found were not strong and not numerous.

### Metal Concentrations

Generally, wild animals show a higher accumulation of pollutants and variation of these in their tissues than farm animals. They may be efficient biomonitors of the current pollution of the ecosystem [26]. The accumulation may be influenced by many factors. Many investigations have revealed no connection between the accumulation and the sex of the animals (e.g., Binkowski et al. 2016), a conclusion borne out in our study, so some researchers have decided to go as far as to omit this factor in the sampling protocol or inference [27–29]. The size of accumulation usually differs between metals. In the case of animals studied, the highest Cd, Cu, and Hg concentrations were observed in kidney tissue. The highest accumulation of metals in kidney tissue was also observed in wild boars from Croatia [26, 30] and northern Poland [31].

Cd concentrations found in our study were medium in comparison to the literature, and higher concentrations had already been observed [30]. We may therefore state that cadmium exposure (generally low) in the habitat was comparable to other areas. On the other hand, when compared to data gathered in Austria, values in the muscle tissue of wild boars hunted in Slovakia were significantly higher [32]. The significantly higher concentrations found in kidney and liver tissue than in muscle tissue are probably connected with the known turnover of Cd in the body where more than 75% of the total burden is deposited in these two organs [18, 33]. We initially suspected that we would observe significantly higher Cd concentrations in older animals, since in many studies, age-related Cd accumulation has been noted [18, 27, 34, 35]. No such trend was encountered in the animals studied, but in particular, the highest Cd concentrations were observed in the oldest animals (similarly to [27]).

We found higher Pb accumulation in muscle tissue than in kidney tissue, which was also mentioned in the literature [26]. Non-outlier maximum and upper quartile concentrations noted in all the organs studied did not exceed 1.0 μg/g w.w., which is not a high value in comparison to other species and areas [27, 36]. This suggests that, in most specimens, pollution did not result from the lead bullet, but other sources of lead were responsible. Apart from the general pollution of the environment (including air, water, sewage, and landfill waste), exposure through the trophic chain was also possible. One of the potential exposure routes for wild boars may be a transfer by the trophic net from Pb poisoned waterfowl [36–38] because in some circumstances, poisoned water birds may be easily hunted by boars, since the animal is an omnivore [15]. Based on the relatively low concentrations found, however, the abovementioned problem did not occur in the specimens studied.

Despite some findings from bird studies, Hg concentrations in the body usually reach the highest values in kidney tissue [27, 31, 39]. Our results confirm this finding with the additional observation that concentrations in kidney and liver tissue are comparable. In general, the contamination of wild animals with Hg in Slovakia is believed to be decreasing [40]. In comparison to Hg concentrations in the liver and kidney tissue of wild boars from northern Poland, the values obtained in the study are slightly lower in kidney tissue, but comparable in liver tissue [31]. Accumulation in muscle tissue was also slightly lower [27]. In comparison to concentrations observed in wild boars shot in the air-polluted Zemplin area (eastern Slovakia), values noted in our studies were significantly lower, for kidney and liver tissue up to seven or eight times lower. Concentrations in muscle tissue were, however, similar [21]. However, concentrations found in the muscle tissue in wild boars from Slovakia were slightly higher than those found in Austria [32]. As our and other research showed, Hg accumulation is not linked with the age of an animal [20].

The other elements studied, Co, Cu, Co, and Zn, belong to the group of essential elements [18]. They are less intensively studied in the literature as xenobiotic metals. The highest median concentrations were noted for Zn, which is consistent with the literature. Similar concentrations in kidney tissue (21.5 μg/g w.w., recalculated from 91.3 μg/g d.w. according to the protocol [41]) were noted in wild boars from southern Poland shot in the late 1980-1989. The concentrations we found in muscle tissue, however, were two times lower (12.1 compared to 30.5 μg/g w.w.) [42]. Studies on red deer revealed that muscle tissue from that species were richer in Zn than liver and kidney tissue was, which is not consistent with our observations [43]. Co and Cu were the only metals studied whose concentrations revealed differences according to age group. The comparison to literature is hard, since similar data is scarce. The categorization of hunted game according to age is problematic because most hunted animals are young [27]. Our observations suggest that accumulation of these metals may be inversely proportional to age. Cu concentrations in the kidney and liver tissue of animals studied were slightly lower in comparison to animals from the north of Poland [27]. Co concentrations revealed no variation between tissues, in contrast to the values noted in red deer from northern Poland. Concentrations in wild boars were also higher [43]. This may be explained by the different position these two species assume in the trophic chain.

Only a few moderately strong correlations were found. The strongest, Cu-Zn in muscle tissue, occurred twice and may be explained by the numerous physiological functions of both elements, including being cofactors of many enzymes [18, 43]. We initially suspected positive correlations of xenobiotic metals, which might suggest the source of both elements in the habitat (e.g., industry) [44]. We observed, however, no such relationship, probably because the concentrations found were generally insufficiently high to lead to intense exposure.

### Fitness for Consumption

Due to the increasing population of wild boar, its meat is widely consumed by people around the world. Both the supply and the quality of the meat (including the flavor, fatty composition, and higher α-tocopherol concentrations) have stimulated the growing demand of the market [45]. Apart from many xenobiotics, metal concentrations in meat and edible organs are also considered regarding its fitness for consumption [19]. Official documents in Europe, however, include no thresholds for game meat and focus mainly on farm-reared animals, with the exception of fish. This is probably the upshot of the historic situation, when game meat was consumed locally only by small numbers of people [46].

Comparing the values obtained with the threshold dedicated to pig meat, we may evaluate only two metals, Cd and Pb (Hg concentrations are regulated only in fish and fishery products). The Cd threshold varies between meat, liver, and kidney tissue and are as follows: 0.05, 0.5, and 1.0 μg/g w.w. [19]. The minimal concentration found in muscle tissue of wild boars examined was only a little lower than the threshold (0.043 μg/g w.w.), but the lower quartile was already higher (0.089 μg/g w.w.), which means that meat from more than 75% specimens studied exceeded the permissible Cd concentrations. The exceedances are less numerous in liver tissue, but still almost half of the specimens had higher concentrations than the threshold. In the case of kidney tissue, only one specimen fell within the permissible range, so the kidney tissue of 98% of the specimens studied were unfit for human consumption. The only possible explanation for the exceedances is the environmental exposure of the animals [46].

The Pb threshold varies only for meat (0.1 μg/g w.w.) and edible organs (0.5 μg/g w.w.) [19]. The thresholds were exceeded in the muscle tissue of 90% of specimens, in the kidney tissue of 28% of specimens and in the liver tissue of 10% of specimens. Here, as well as environmental pollution ammunition also plays a role. The common problem of Pb concentrations in game meat is linked with Pb shots and bullet fragments. Such Pb transfer depends on the type of bullet, its mushrooming abilities, the size of the animals, and the tissue hit. Tissue 25–30 cm or even further away from the wound may still contain small Pb particles [47, 48]. Other research showed that the Pb transfer is more efficient in cases in which lead pellet ammunition (the ammunition more commonly used in hunting small game, such as waterfowl, hares, and foxes) was used rather than bullets (the ammunition more commonly used in hunting big game). The problem of lead transfer cannot be solved without banning the use of Pb bullets, but the resistance of the hunting community is considerable. Hunters commonly think that the lead-free ammunition is inefficient from a ballistic point of view (e.g., they are prone to injuring animals rather than killing them). This is still questionable, but scientific research shows that the efficiency is not dependent on whether the ammunition is lead or lead-free, but rather the type of bullet [49, 50].

Despite the established permissible content in food and provisional tolerable weekly intake (PTWI), some organizations postulate that all Pb should be eradicated because of its high toxicity according to the ALARA approach (As Low As Reasonably Achievable) [19, 51, 52]. Pb PTWI is especially believed to be no longer appropriate and should be lowered. Thus, we assessed the risk to consumers of Pb from wild boar meat on the basis of the new protocol proposed by European Food Safety Agency (EFSA) [51]. This attempt is based on the margin of exposure value (MOE) calculated as the ratio between a defined point on the dose-response curve for the adverse effect (the benchmark dose lower confidence limit) and estimated intake with food. According to EFSA’s opinion, a MOE value of 10 or greater ensures that there is no appreciable risk of a clinically significant change in the prevalence of chronic kidney disease (CKD) and an increase in systolic blood pressure (SBP). The risk of a MOE of greater than 1.0 is very low [51]. We may say then that the consumption of the wild boar meat studied poses no special risk of SBP to any consumer when we discuss the average concentrations (medians). For samples containing maximum concentrations, the risk is high, especially for high and extreme consumption groups (Table 4). Similar results were obtained for CKD, but here, the risk calculated in every group is higher even for average concentrations found. It should be stressed that MOE values only indicate a level of concern and do not quantify risk [54].

In the case of Cd, the simpler PTWI approach is still common. The exceedances of PTWI calculated on the basis of concentrations found in animals studied are common: only muscle tissue containing average concentrations seems to be safe for human consumption where Cd is concerned (Table 5).

**Table 5:** Simulation of the consumer exposure with reference to Cd concentrations found in the tissue studied according to the provisional tolerable weekly intake (PTWI) calculated for 70 kg person: 175 μg

Concentration used (μg/g w.w.)	Weight of the total portion (100% PTWI)	No. of 200 g portions	Contribution of 4 meals to PTWI (%)	Total weekly intake (% PTWI)
Muscle (Q_2_ 0.155)	1129	5.6	70.9	119–211
Muscle (max. 0.373)	469	2.3	171	219–311
Liver (Q_2_ 0.474)	369	1.8	217	265–357
Liver (max. 1.92)	91.1	0.5	878	926–1018
Kidneys (Q_2_ 2.73)	64.1	0.3	1249	1297–1389
Kidneys (max. 8.82)	19.9	0.1	4030	4078–4170

Simulation calculated for median (Q_2_) and maximum value noted (max.). Initial PTWI value is 2.5 μg/g body weight [55]. Total weekly intake includes 4 meals of product studied, general weekly intake taken from literature 48–140% [46, 56]

**Table 4:** Estimated MOEs for different endpoints by the intensity of game consumption

Concentration used [μg/g w.w.]	MOE–normal consumption	MOE–high consumption	MOE–extreme consumption
Cardiovascular effects			
Muscle (Q_2_ 0.1550)	1.2–4.1	1.2–3.7	0.9–2.1
Muscle (Max. 0.3732)	0.6–1.0	0.2	0.03
Nephrotoxicity			
Muscle (Q_2_ 0.1550)	0.5–1.7	0.5–1.6	0.4–0.9
Muscle (Max. 0.3732)	0.3–0.4	0.1	0.01

MOE (margin of exposure) values calculated for normal consumption (2 game meals per year), high consumption (10 game meals per year) and extreme consumption (90 game meals per year) [53]

Concentrations of other metals studied are currently not officially regulated by law or norms [19]. Older documents from various countries, however, present the then permissible values for canned meat products. In Poland, for instance, the threshold in the 1980s was 8.0 and 50.0 mg/kg for Cu and Zn, respectively [57]. According to them, all the samples from our study fell within the permissible range of Cu and Zn concentrations.

## Conclusions

Concentrations found in the study relative to literature data seem not to be increased. During the literature comparison, we observed no significant trends in time, but only rather a moderate variation in concentrations between areas. Some metals in the study, such as Cd and Pb, however, reached concentrations sufficiently high to exceed the permissible accumulation in meat (thresholds are only established for farm-reared meat) and to increase the risk of the consumers. It should be stressed here that Cd and Pb concentrations were not correlated and thus, only one specimen which had safe Cd concentrations in kidney tissue exceeded concentrations of Pb in muscle tissue, so none of the animals studied was fully fit for human consumption. This observation imposes questions about the usefulness of the thresholds intended for farm-reared animals for game meat, and also about the necessity of the regular monitoring of selected metallic pollutants in hunted animals.

## References

[1] Bjerregaard P, Johansen P, Mulvad G (2004). Lead sources in human diet in Greenland. Environ Health Perspect.

[2] Johansen P, Asmund G, Riget F (2004). High human exposure to lead through consumption of birds hunted with lead shot. Environ Pollut.

[3] Szmańko T, Górecka J, Korzeniowska M (2007). Comparison of chosen quality parameters of meat from wild boar and domestic pigs. Polish J Food Nutr Sci.

[4] Klein PN (2004) Game meat: a complex food safety and animal health issue. Food Saf Mag 1–6

[5] Shore RF, Casulli A, Bologov V (2001). Organochlorine pesticide, polychlorinated biphenyl and heavy metal concentrations in wolves (*Canis lupus* L. 1758) from north-west Russia. Sci Total Environ.

[6] Kramárová M, Massányi P, Slamecka J (2005). Distribution of cadmium and lead in liver and kidney of some wild animals in Slovakia. J Environ Sci Heal Part A.

[7] Gasparik J, Massanyi P, Slamecka J (2004). Concentration of selected metals in liver, kidney, and muscle of the red deer (*Cervus elaphus*). J Environ Sci Heal Part A.

[8] Kolesarova A, Slamecka J, Jurcik R (2008). Environmental levels of cadmium, lead and mercury in brown hares and their relation to blood metabolic parameters. J Environ Sci Heal Part A.

[9] Gasparik J, Vladarova D, Capcarova M (2010). Concentration of lead, cadmium, mercury and arsenic in leg skeletal muscles of three species of wild birds. J Environ Sci Heal Part A.

[10] Abbasi NA, Jaspers VLB, Chaudhry MJI (2015). Influence of taxa, trophic level, and location on bioaccumulation of toxic metals in bird’s feathers: a preliminary biomonitoring study using multiple bird species from Pakistan. Chemosphere.

[11] Markov NI, Neifel ND, Estafev AA (2004). Ecological aspects of dispersal of the wild boar, *Sus scrofa* L., 1758, in the northeast of European Russia. Russ J Ecol.

[12] Sáez-Royuela C, Tellería JL (1986). The increased population of the wild boar (*Sus scrofa* L.) in Europe. Mamm Rev.

[13] Schley L, Roper TJ (2003). Diet of wild boar *Sus scrofa* in Western Europe, with particular reference to consumption of agricultural crops. Mamm Rev.

[14] Cahill S, Llimona F, Gràcia J (2003). Spacing and nocturnal activity of wild boar *Sus scrofa* in a Mediterranean metropolitan park. Wildlife Biol.

[15] Giménez-Anaya A, Herrero J, Rosell C (2008). Food habits of wild boars (*Sus scrofa*) in a mediterranean coastal wetland. Wetlands.

[16] Geisser H, Reyer HU (2004). Efficacy of hunting, feeding, and fencing to reduce crop damage by wild boars. J Wildl Manag.

[17] Baubet E, Bonenfant C, Brandt S (2004). Diet of the wild boar in the French Alps. Galemys.

[18] Nordberg GF, Fowler BA, Nordberg M, Friberg LT (2007). Handbook on the toxicology of metals.

[19] EC (2006). 1881/2006. Setting maximum levels for certain contaminants in foodstuffs. Off J Eur Communities.

[20] Srebočan E, Crnić AP, Ekert-Kabalin AM (2011). Cadmium, lead, and mercury concentrations in tissues of roe deer (*Capreolus capreolus* L.) and wild boar (*Sus scrofa* L.) from lowland Croatia. Czech J Food Sci.

[21] Piskorová L, Vasilková Z, Krupicer I (2003). Heavy metal residues in tissues of wild boar (*Sus scrofa*) and red fox (*Vulpes vulpes*) in the Central Zemplin region of the Slovak Republic. Czech J Anim Sci.

[22] Santiago D, Motas-Guzmán M, Reja A (1998). Lead and cadmium in red deer and wild boar from Sierra Morena Mountains (Andalusia, Spain). Bull Environ Contam Toxicol.

[23] Suran J, Prisc M, Rasic R (2013). Malondialdehyde and heavy metal concentrations in tissues of wild boar (*Sus scrofa* L.) from central Croatia. J Environ Sci Heal Part B.

[24] Yarsan E, Yipel M, Dikmen B (2014). Concentrations of essential and non-essential toxic trace elements in wild boar (*Sus scrofa* L., 1758) tissues from southern Turkey. Bull Environ Contam Toxicol.

[25] Quinn GP, Keough MJ (2002). Experimental design and data analysis for biologists.

[26] Bilandžić N, Sedak M, Crossed D, Signokić M, Šimić B (2010). Wild boar tissue levels of cadmium, lead and mercury in seven regions of continental Croatia. Bull Environ Contam Toxicol.

[27] Falandysz J (1994). Some toxic and trace metals in big game hunted in the northern part of Poland in 1987–1991. Sci Total Environ.

[28] Reglero M, Taggart MA, Monsalve-González L, Mateo R (2009). Heavy metal exposure in large game from a lead mining area: effects on oxidative stress and fatty acid composition in liver. Environ Pollut.

[29] Crnić AP, Šuran J, Madunić HC, Božić F (2015). Cadmium concentrations in the tissues of young wild boar (*Sus scrofa* L.) from Moslavina and Slavonia in lowland Croatia. Vet Arh.

[30] Bilandžić N, Sedak M, Vratarić D (2009). Lead and cadmium in red deer and wild boar from different hunting grounds in Croatia. Sci Total Environ.

[31] Dobrowolska A, Melosik M (2002). Mercury contents in liver and kidneys of wild boar (*Sus scrofa*) and red deer (*Cervus elaphus*). Z Jagdwiss.

[32] Ertl K, Kitzer R, Goessler W (2016). Elemental composition of game meat from Austria. Food Addit Contam Part B.

[33] Gunn SA, Gould TC (1957). Selective accumulation of Cd115 by cortex of rat kidney. Proc Soc Exp Biol Med.

[34] Scheuhammer AM (1987). The chronic toxicity of aluminium, cadmium, mercury and lead in birds: a review. Environ Pollut.

[35] Binkowski ŁJ, Sawicka-Kapusta K (2015). Cadmium concentrations and their implications in Mallard and Coot from fish pond areas. Chemosphere.

[36] Binkowski ŁJ, Sawicka-Kapusta K (2015). Lead poisoning and its in vivo biomarkers in Mallard and Coot from hunting activity areas. Chemosphere.

[37] Scheuhammer AM, Norris SL (1996). The ecotoxicology of lead shot and lead fishing weights. Ecotoxicology.

[38] Pain DJ, Matthews G (1990). Lead poisoning of waterfowl: a review. IWRB Symp.

[39] Aazami J, Esmaili-Sari A, Bahramifar N, Savabieasfahani M (2012). Total and organic mercury in liver, kidney and muscle of waterbirds from wetlands of the Caspian Sea, Iran. Bull Environ Contam Toxicol.

[40] Zmetáková Z, Šalgovicova D (2008) Contamination of the wild animals with mercury in the Slovak Republic. In: Bezpečnosť a Kval. surovín a potravín, III. Nitra, pp 1–5

[41] Binkowski ŁJ (2012). The effect of material preparation on the dry weight used in trace elements determination in biological samples. Fresenius Environ Bull.

[42] Świergosz R, Perzanowski K, Makosz U, Biłek I (1993). The incidence of heavy metals and other toxic elements in big game tissues. Sci Total Environ.

[43] Jarzyńska G, Falandysz J (2011). Selenium and 17 other largely essential and toxic metals in muscle and organ meats of red deer (*Cervus elaphus*)—consequences to human health. Environ Int.

[44] Karimi M-HS, Hassanpour M, Pourkhabbaz A-R (2016). Trace element concentrations in feathers of five Anseriformes in the south of the Caspian Sea, Iran. Environ Monit Assess.

[45] Sales J, Kotrba R (2013). Meat from wild boar (*Sus scrofa* L.): a review. Meat Sci.

[46] Taggart MA, Reglero MM, Camarero PR, Mateo R (2011). Should legislation regarding maximum Pb and Cd levels in human food also cover large game meat?. Environ Int.

[47] Dobrowolska A, Melosik M (2008). Bullet-derived lead in tissues of the wild boar (*Sus scrofa*) and red deer (*Cervus elaphus*). Eur J Wildl Res.

[48] Hunt WG, Watson RT, Oaks JL (2009). Lead bullet fragments in venison from rifle-killed deer: potential for human dietary exposure. PLoS One.

[49] Gremse F, Krone O, Thamm M (2014). Performance of lead-free versus lead-based hunting ammunition in ballistic soap. PLoS One.

[50] Trinogga A, Fritsch G, Hofer H, Krone O (2013). Are lead-free hunting rifle bullets as effective at killing wildlife as conventional lead bullets? A comparison based on wound size and morphology. Sci Total Environ.

[51] EFSA (2010). Scientific opinion on lead in food. EFSA J.

[52] WHO (2000). Evaluation of certain food additives and contaminants.

[53] BfR (2014) Safety of game meat obtained through hunting

[54] EFSA (2012). Statement on the applicability of the margin of exposure approach for the safety assessment of impurities which are both genotoxic and carcinogenic in substances added to food/feed. EFSA J.

[55] EFSA (2009). Cadmium in food. EFSA J.

[56] Nasreddine L, Parent-Massin D (2002) Food contamination by metals and pesticides in the European Union. Should we worry? In: Toxicol. Lett. pp 29–41 10.1016/s0378-4274(01)00480-5 12052638

[57] MZiOS (1985) Zarządzenie MInistra Zdrowia i Opieku Społecznej w sprawie wykazu substancji dodatkowych dozwolonych i zanieczyszczeń technicznych w środkach spożywczych i używkach oraz na ich powierzchni. pp 469–492

[58] Binkowski ŁJ, Merta D, Przystupińska A (2016). Levels of metals in kidney, liver and muscle tissue and their relation to the occurrence of parasites in the red fox in the Lower Silesian Forest in Europe. Chemosphere.

